# Foxj1 expressing ependymal cells do not contribute new cells to sites of injury or stroke in the mouse forebrain

**DOI:** 10.1038/s41598-018-19913-x

**Published:** 2018-01-29

**Authors:** Nagendran Muthusamy, Andrew Brumm, Xuying Zhang, S. Thomas Carmichael, H. Troy Ghashghaei

**Affiliations:** 1 WM Keck Center for Behavioral Biology, Program in Genetics, Program in Comparative Biomedical Sciences, Department of Molecular Biomedical Sciences, College of Veterinary Medicine, North Carolina State University,, 1060 William Moore Dr., Raleigh, NC 27607 USA; 2 Department of Neurology, David Geffen School of Medicine, University of California, Los Angeles, 635 Charles Young Drive, CA 90095 USA

## Abstract

The stem cell source of neural and glial progenitors in the periventricular regions of the adult forebrain has remained uncertain and controversial. Using a cell specific genetic approach we rule out Foxj1+ ependymal cells as stem cells participating in neurogenesis and gliogenesis in response to acute injury or stroke in the mouse forebrain. Non stem- and progenitor-like responses of Foxj1+ ependymal cells to injury and stroke remain to be defined and investigated.

## Introduction

Past reports have suggested that adult ependymal cells (ECs), or a subpopulation thereof, have endogenous stem cell potential with the ability to generate new neurons for the olfactory bulbs (OBs) and in response to stroke in the mouse forebrain. In one study, intraventricular injections of adeno or lenti viruses driving expression of reporters downstream of the human *FOXJ1* promoter resulted in labeling of new cells generated from transduced cells only after induction of stroke but not in naïve adult mice^1^. The same human promoter element was used in a subsequent study leading the authors to postulate substantial plasticity in the EC lineage and their relationship to nearby astrocytes^2^. The same promoter was also cloned into a reporter *piggyback* vector and electroporated into the rat brain, resulting in lineage-traced cells in the olfactory bulbs after 6 or 12 weeks in both healthy and stroke-induced brains through medial cerebral artery occlusions (MCAO)^3^. In other studies of the spinal cord, similar lineage tracing approaches were utilized to show that a substantial portion of the glial scar in damaged spinal cords come from ECs^4–6^ presumably due to their extensive proliferation^7^.

Concerned that the human promoter element utilized in the past studies (a ~ 1 kb upstream human *FOXJ1* locus) was resulting in ectopic expression patterns, we generated a knock-in *Foxj1*^*creERT2::GFP*^ mouse to lineage-trace potential EC progeny from the endogenous locus. This line has been characterized^8^ and was used in a recent study illustrating that spinal cord injury fails to induce Foxj1+ ECs to proliferate or to substantially contribute new cells to the glial scar^9^. To test the possibility that damage or stroke in the forebrain may contribute to the reported transformation of ependyma into neurogenic or gliogenic progenitors, a stab injury and three distinct stroke models were employed.

## Results

**Figure 1:**
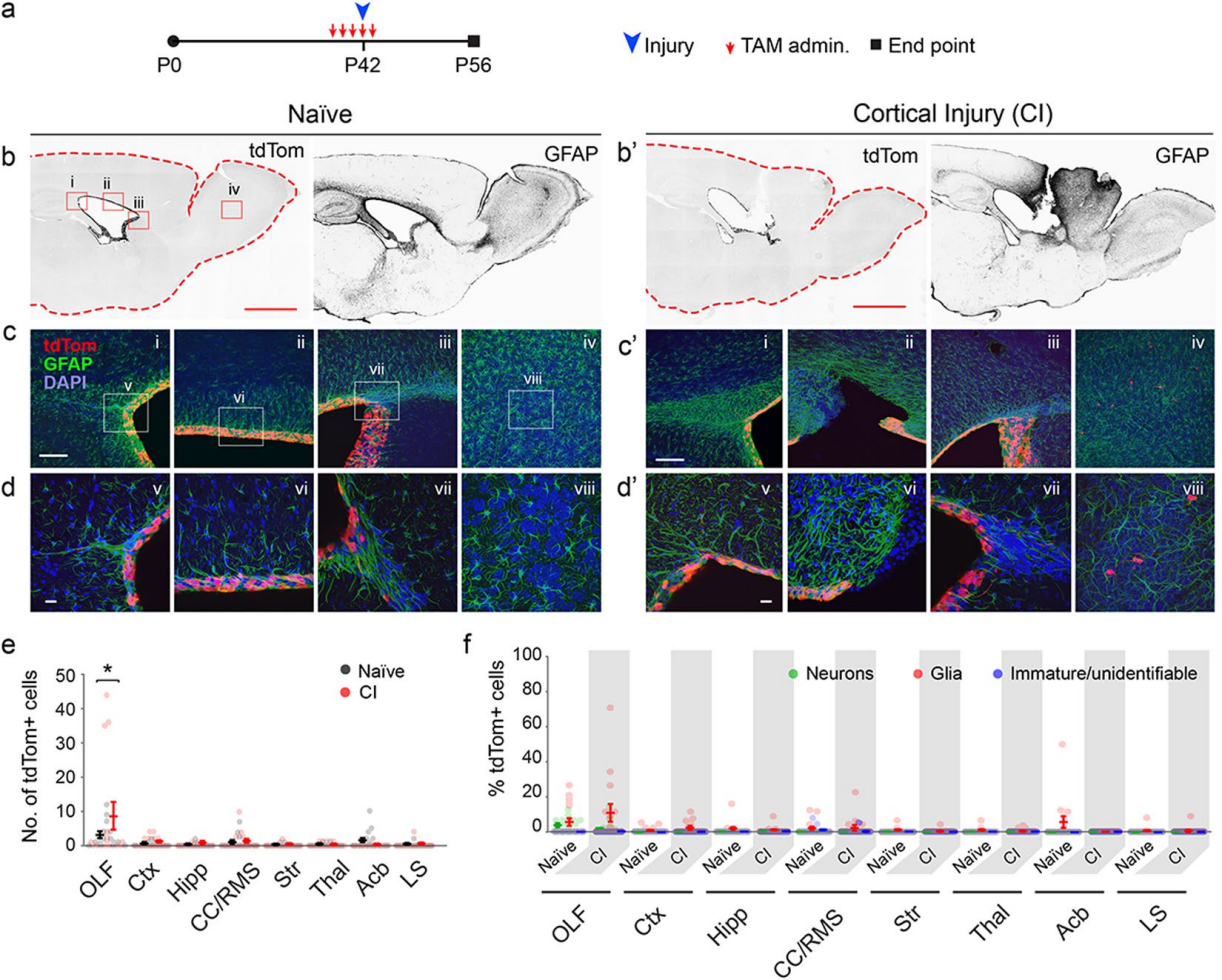
Lack of EC expansion or cellular contribution to sites of cortical injury. (**a**) Experimental paradigm for initiation of recombination in ECs, timing of cortical injury, and analysis. (**b/b’**) Low magnification images of tdTom and GFAP signals in forebrains of Naïve and cortically injured mice (fluorescent signals were rendered to black for visualization). Scale bar: 500 µm. Boxed areas in tdTom image under ‘Naïve’ represent areas magnified in (**c/c’**) (i–iv). (**c/c’**–**d/d’**) Higher magnification images of regions demarcated in (**b/b’**) of the caudal SEZ (i, v), dorsal SEZ (ii, vi), rostral SEZ (iii, vii), and OB (iv, viii). Scale bars: (**c/c**’), 50 µm; **d/d’**, 10 µm. (**e**) Quantification of cell numbers in forebrain regions of naïve and experimental mice in which sporadic cells were found (n = 5 sagittal sections per each of three mice per group). Data points are from individual sagittal forebrain sections. Error bars are for mean ± s.e.m.; Significance (*) was determined at p < 0.05, *Student’s t test*. (**f**) Identification of cells in forebrain regions of naïve and cortical injury (CI) mice as neurons, glia or immature cells (see Fig. 2 for morphological characterizations). Data points are from individual sagittal forebrain sections. Error bars are for mean ± s.e.m. Note on instances where the tdTom+ EC layer appears multilayered: the 50 µm thickness of sections when mounted results in flattening of the surface of the ventricles and a mirage of multilayered ECs.

**Figure 2:**
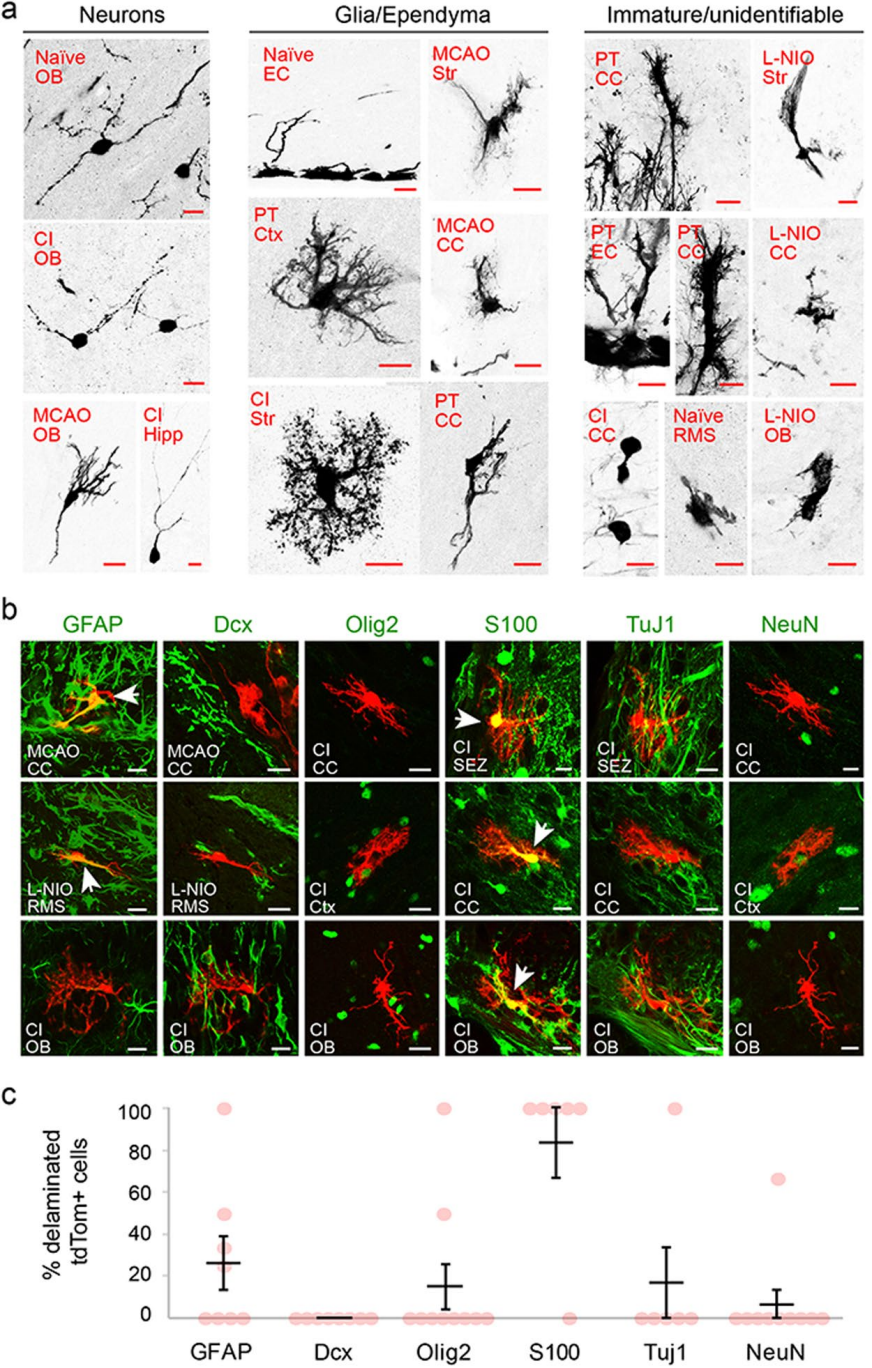
Morphological characterization of neurons, glial and immature/unidentified cells in the forebrains of naïve and experimental mice. (**a**) Images are of tdTom+ cells rendered to monochrome for ease of visualization. Labels on each image indicate injury or stroke model (top row) and region from which the cell was captured (bottom row). (**b**) Marker analysis of the sparse tdTom+ cells found within the scar or infaract regions of injury and stroke models. GFAP, Olig2, Dcx label reactive astrocytes, glial progenitors and neuroblasts, respectively. Note, little to no overlap between these markers and the tdTom+ cells. Scale bars, 10 um. (**c**) Percentages of delaminated tdTom+ cells in mice with cortical injury, positive for various markers: GFAP, reactive astrocytes; Dcx, neuroblasts; Olig2, glia progenitors; S100, protoplasmic astrocytes; Tuj1, immature neurons; NeuN, mature neurons. Data points are from individual sagittal forebrain sections; n = 3 animals.

Recombination was induced by tamoxifen administration (TAM) in naïve and experimental mice, and cre-dependent expression of tdTomato (tdTom) was quantified using the well-established Ai9 reporter allele. In experimental animals, TAM was administered daily at postnatal day 39 (P39) for five days in young adult mice, stab injuries were inflicted in the motor cortex on the forth day of TAM administration (at P42), followed by perfusion and analysis two weeks later at P56 (Fig. 1a). Two weeks post-injury is a well-established time line for neurogenic and gliogenic responses to injury and stroke based on numerous past studies^10–12^. Sectioning and microscopic analysis of each brain revealed little to no tdTom+ cells anywhere along the injured site or in surrounding forebrain regions (Fig. 1b/b’). The scarce tdTom+ cells near the site of injury were found within the scar tissue as revealed by GFAP staining (Fig. 1c/c’), and were nearly all glia-like (Fig. 2a). In addition, there was a slight, yet significant elevation in the number of cells found in the OBs of injured brains (Fig. 1d,e), with most of them resembling immature neurons (Figs 1f and 2a). Analysis with established markers for neurons and glia revealed robust overlap of the rare delaminated tdTom+ cells (those outside the ependymal layer) with the glial marker S100, and far less with GFAP or Olig2 within and around the scar region in the subependymal zone (SEZ), white matter (WM) or cortical parenchyma (Ctx) overlying the ventricles (Fig. 2b,c).

**Figure 3:**
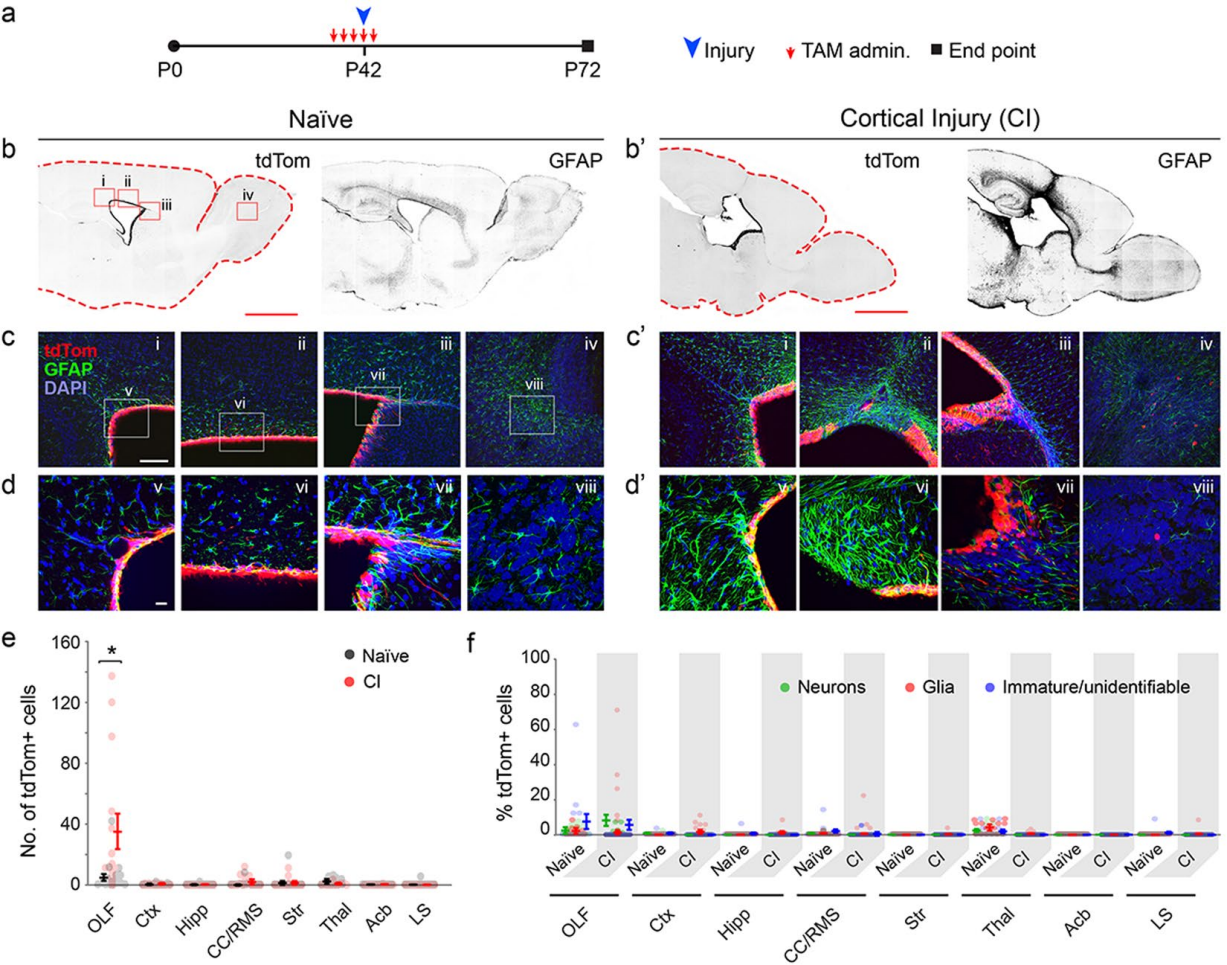
Lack of EC expansion or cellular contribution to sites of cortical injury after prolonged survival. (**a**) Experimental paradigm for initiation of recombination in ECs, timing of cortical injury, and analysis. (**b/b’**) Low magnification images of tdTom and GFAP signals in forebrains of Naïve and cortically injured mice (fluorescent signals are rendered to black for visualization). Scale bar: 500 µm. Boxed areas in tdTom image under ‘Naïve’ represent areas magnified in (**c/c’**) (i–iv). (**c/c’**–**d/d’**) Higher magnification images of regions demarcated in (**b/b’**) of the caudal SEZ (i, v), dorsal SEZ (ii, vi), rostral SEZ (iii, vii), and OB (iv, viii). Scale bars: (**c/c’**), 50 µm; d/d’, 10 µm. (**e**) Quantification of cell numbers in forebrain regions of naïve and experimental mice in which sporadic cells were found (n = 5 sagittal sections per each of three mice per group). Data points are from individual sagittal forebrain sections. Data are mean ± s.e.m.; Significance (*) was determined at p < 0.05, Student’s t test. (**f**) Identification of cells in forebrain regions of naïve and cortical injury (CI) mice as neurons, glia or immature cells (see Fig. 2 for morphological characterizations). Data points are from individual sagittal forebrain sections. Data are mean ± s.e.m. Note on instances where the tdTom+ EC layer appears multilayered: the 50 µm thickness of sections when mounted results in flattening of the surface of the ventricles and a mirage of multilayered ECs.

**Figure 4:**
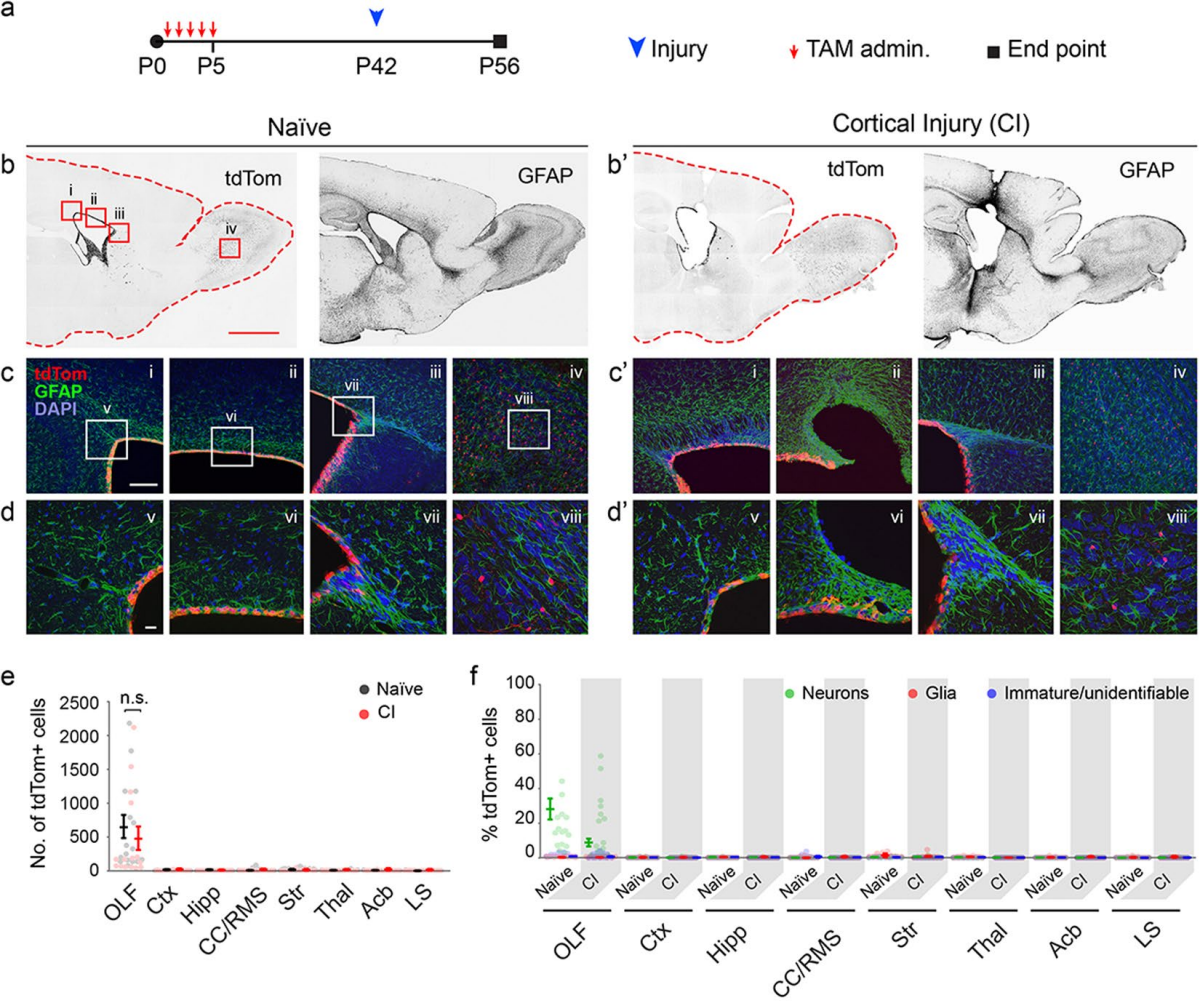
Lack of EC expansion or cellular contribution to sites of cortical injury after induction during early postnatal days. (**a**) Experimental paradigm for initiation of recombination in ECs from P1 to P5, timing of stroke induction, and analysis. (**b/b’**) Low magnification images of tdTom and GFAP signals in forebrains of Naïve and cortically injured mice (fluorescent signals were rendered to black for visualization). Scale bar: 500 µm. Boxed areas in tdTom image under ‘Naïve’ represent areas magnified in (**c/c’**) (i–iv). (**c/c’**–**d/d’**) Higher magnification images of regions demarcated in (**b/b’**) of the caudal SEZ (i, v), dorsal SEZ (ii, vi), rostral SEZ (iii, vii), and OB (iv, viii). Scale bars: (**c/c’**) 50 µm; (**d/d’**) 10 µm. (**e**) Quantification of cell numbers in forebrain regions of naïve and experimental mice in which sporadic cells were found (n = 5 sagittal sections per each of 3 mice per group). Data points are from individual sagittal forebrain sections. Error bars are for mean ± s.e.m.; Significance (*) was determined at p < 0.05, *Student’s t test*. (**f**) Identification of cells in forebrain regions of naïve (N) and cortical injury (CI) mice as neurons, glia or immature cells (see Fig. 2 for morphological characterizations). Data points are from individual sagittal forebrain sections. Error bars are for mean ± s.e.m. Note on instances where the tdTom+ EC layer appears multilayered: the 50 µm thickness of sections when mounted results in flattening of the surface of the ventricles and a mirage of multilayered ECs.

To assess whether prolonged survival post injury would recruit a potential slow-responsive ependymal progenitor pool to contribute cells to the injured site, we extended survival of P42 induced/P46 injured mice to 1-month post injury. Prolonged survival failed to capture any additional tdTom+ cells within the forebrain (Fig. 3). Moreover, since we consistently find some tdTom+ neurons in the OB when recombination is induced between P0-P21 in *Foxj1*^*creERT2*^ mice^8^, we wondered if a quiescent population of Foxj1+ ECs may only be genetically accessible during early postnatal periods. To test this possibility, early postnatal-induced mice (TAM administered P1-P5) were injured in their motor cortex at P42 (Fig. 4). Again, little to no tdTom+ cells were found within or surrounding the site of injury when analyzed at P56 (Fig. 4). As expected, and in contrast to P39-P44 induced brains, there were substantially higher number of cells in the OBs (Fig. 4e) confirming our past findings, with most of them resembling neurons (Fig. 4f). However, there was no significant difference in number or types of cells in the OBs of experimental and naïve mice, suggesting no proliferative or differentiative response by Foxj1-derived cells to cortical injury. These findings suggest that ECs for the most part fail to participate in generation of new cells within sites of injury in the forebrain, as also recently reported in the spinal cord^3^.

**Figure 5:**
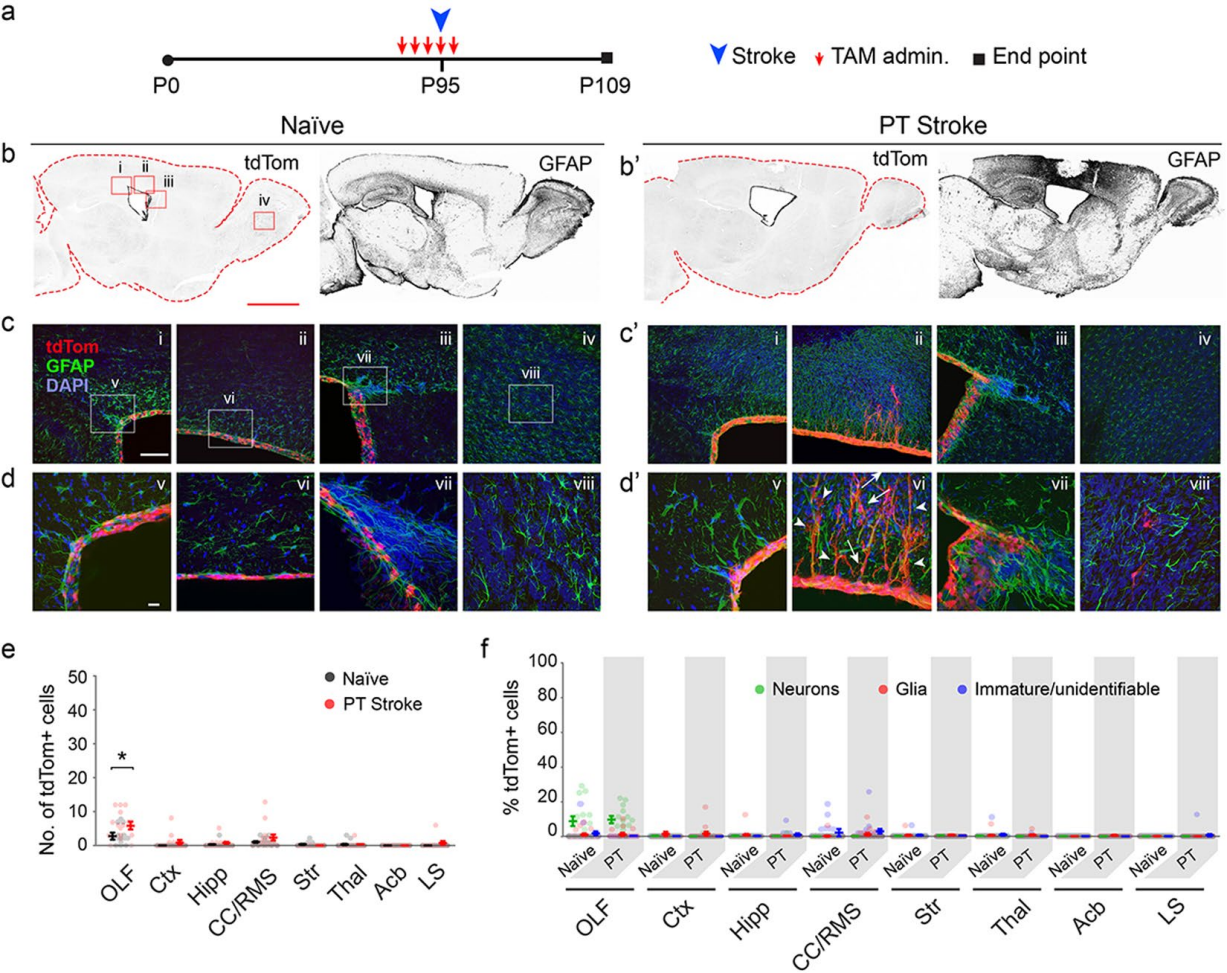
Lack of EC expansion or cellular contribution in PT-induced cortical stroke. (**a**) Experimental paradigm for initiation of recombination in ECs, timing of stroke induction, and analysis. (**b/b’**) Low magnification images of tdTom and GFAP signals in forebrains of Naïve and cortically injured mice (fluorescent signals were rendered to black for visualization). Scale bar: 500 µm. Boxed areas in tdTom image under ‘Naïve’ represent areas magnified in (**c/c’**) (i-iv). (**c/c’**–**d/d’**) Higher magnification images of regions demarcated in (**b/b’**) of the caudal SEZ (i, v), dorsal SEZ (ii, vi), rostral SEZ (iii, vii), and OB (iv, viii). Scale bars: (**c/c’**) 50 µm; (**d/d’**) 10 µm. Arrows point to delaminated cells in various regions; arrowheads, EC process extensions into the white matter overlying the ventricles. (**e**) Quantification of cell numbers in forebrain regions of naïve and experimental mice in which sporadic cells were found (n = 5 sagittal sections per each of 3 mice per group). Data points are from individual sagittal forebrain sections. Error bars are for mean ± s.e.m.; Significance (*) was determined at p < 0.05, *Student’s t test*. (**f**) Identification of cells in forebrain regions of naïve (N) and cortical injury (CI) mice as neurons, glia or immature cells (see Fig. 2 for morphological characterizations). Data points are from individual sagittal forebrain sections. Error bars are for mean ± s.e.m. Note on instances where the tdTom+ EC layer appears multilayered: the 50 µm thickness of sections when mounted results in flattening of the surface of the ventricles and a mirage of multilayered ECs.

**Figure 6:**
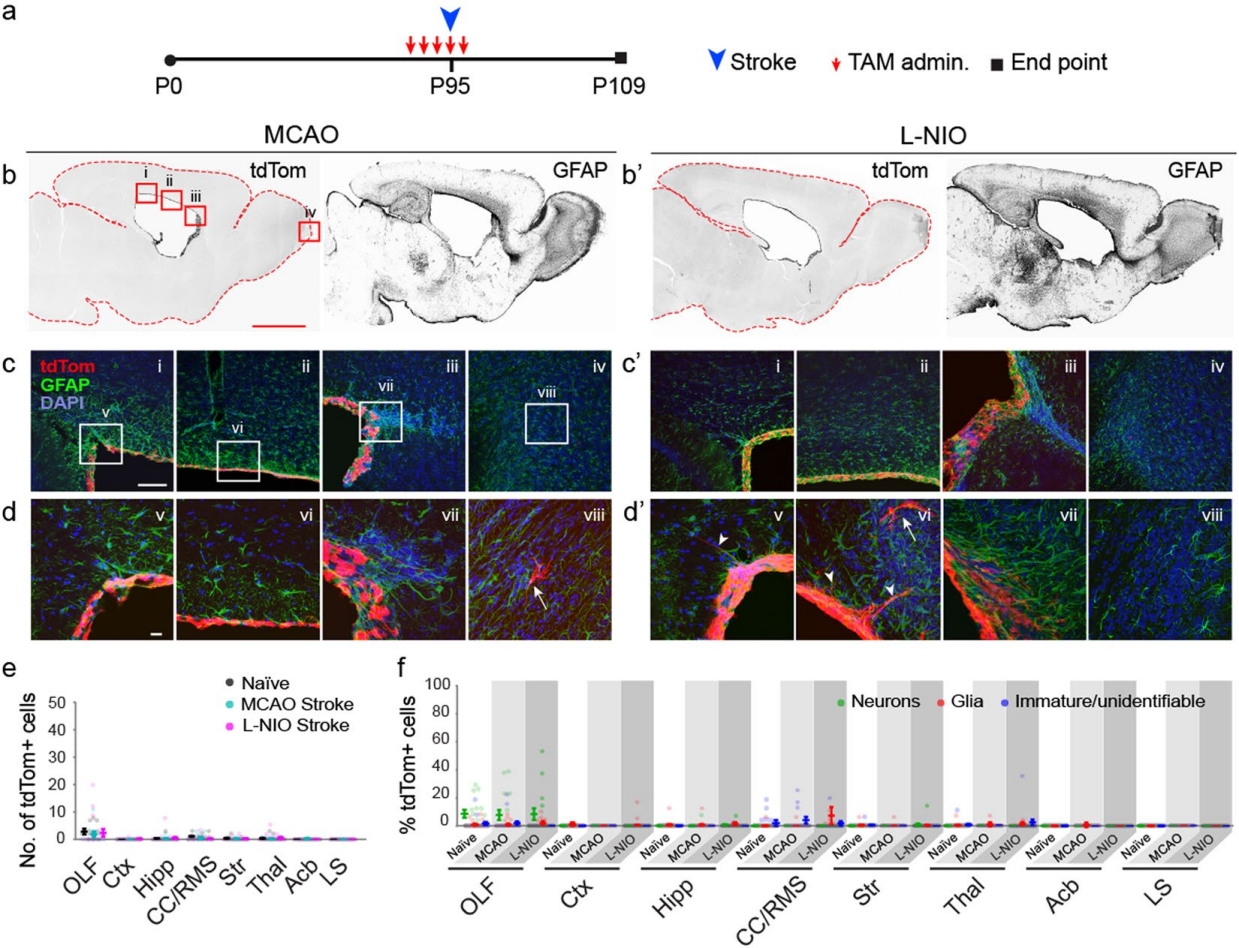
Lack of EC expansion or cellular contribution to sites of infarct after MCAO and L-NIO induced striatal stroke. (**a**) Experimental paradigm for initiation of recombination in ECs in P95 mice, timing of MCAO and L-NIO, and analysis. (**b/b’**) Low magnification images of tdTom and GFAP signals in forebrains of MCAO and L-NIO mice (fluorescent signals were rendered to monochrome for ease of visualization). Scale bar: 500 µm. Boxed areas in tdTom image under ‘MCAO’ represent areas magnified in c/c’ (i-iv). (**c/c’-d/d’**) Higher magnification images of regions demarcated in (**b/b’**) of the caudal SEZ (i, v), dorsal SEZ (ii, vi), rostral SEZ (iii, vii), and OB (iv, viii). Scale bars: (**c/c’**) 50 µm; (**d/d’**) 10 µm. Arrows point to delaminated cells in various regions; arrowheads, EC process extensions into the white matter overlying the ventricles. (**e**) Quantification of cell numbers in forebrain regions of MCAO and L-NIO mice in which sporadic cells were found (n = 5 sagittal sections per each of 3 mice per group); data from naïve mice are also included for comparison. Data points are from individual sagittal forebrain sections. Error bars are for mean ± s.e.m.; Significance (*) was determined at p < 0.05, *Student’s t test*. (**f**) Identification of cells in forebrain regions of naïve (N) and cortical injury (CI) mice as neurons, glia or immature cells (see Fig. 2 for morphological characterizations). Data points are from individual sagittal forebrain sections. Error bars are for mean ± s.e.m. Note on instances where the tdTom + EC layer appears multilayered: the 50 µm thickness of sections when mounted results in flattening of the surface of the ventricles and a mirage of multilayered ECs.

Next, to determine whether stroke transforms ependyma to gain progenitor properties as suggested in a prior study^1^, three models were used; photothrombotic (PT) induced stroke in the motor cortex (Fig. 5), as well as middle cerebral artery occlusion (MCAO) and vasoconstrictive agent N5-(1-iminoethyl)-L-ornithine (L-NIO) induced striatal stroke (Fig. 6). The three models produce damage in three distinct forebrain regions; motor cortex, somatosensory cortex and striatum, which are all associated with neurogenesis after stroke^13,14^. In all three models, P95 *Foxj1*^*creERT2*^ mice were subjects of a stroke induction with 5-day TAM administrations the same as the injury model described above, followed by 14 days of survival (Figs 5a and 6a). Similar to blunt stab injuries, few if any tdTom+ cells were found outside of the EC layer in any of the stroke models applied to TAM-induced *Foxj1*^*creERT2*^ mice. Interestingly, a few tdTom+ cells were delaminated from the EC layer into the overlying WM in the PT model (Fig. 5d’), but these were highly infrequent, and most cells remained within the white matter directly ventral to the site of stroke. There was a profound transformation in the shape of presumptive tdTom+ ECs near these sites exhibiting elongated processes toward the site of stroke (Fig. 5c/c’–d/d’). The delaminated cells did not resemble neurons or astrocytes. Interestingly, we did not observe these transformations in any of the mice inflicted with blunt injury suggesting possible differences in mechanisms that drive responses to trauma versus stroke-associated ischemia in the forebrain.

## Discussion

Taken together, our results indicate minimal, if any, direct cellular contribution from the Foxj1+ ependymal cell pool to sites of insult or other forebrain regions after injury and stroke. These findings are consistent with the recent report on responses of Foxj1+ ECs in the spinal canal to injury^9^. At this juncture, we postulate that the human *FOXJ1* promoter element was most likely the source of mislabeling in the past findings. In fact, two lines of transgenic mice driving EGFP and creERT2 expression under the same human FOXJ1 promoter exhibit far more expression outside the EC layer than the knock-in line employed in the current study. It is possible that a population of Foxj1-negative ECs will exhibit progenitor capacity, thus escaping our lineage tracing method. However, this is highly unlikely since the EC phenotype is unequivocally dependent on Foxj1 expression^15,16^. Moreover, characterization of recombination rate with our tamoxifen induction protocol labels the vast majority of ECs^8^. Even though the current findings rule out Foxj1-expressing ependyma as a source of new cells in response to injury and stroke, the role of ECs in homeostasis of the brain and their cellular responses to injury, disease, and aging are beginning to be deciphered^17,18^.

## Methods

### Animals

Use of mice was in accordance with the US National Institutes of Health Animal Protection Guidelines and approval from institutional animal care and use committees at North Carolina State University and University of California Los Angeles. Heterozygous *Foxj1*^*creERT2*^ mice (Jackson Laboratory, Stock No: 027012) on the Ai9 reporter background (Jackson Laboratory, Stock No: 007909) were generated as described before^8^. Mice were experimentally naïve prior to the studies, were housed at four mice per cage, and maintained on a 12 h light/dark cycle. Expression of tdTomato (tdTom) was induced in adult *Foxj1*^*creERT2*^*/Ai9* mice using a five-day tamoxifen (TAM) induction protocol. Animals were induced at various postnatal time points through intraperitoneal injections of 4-OH TAM (10 mg/ml in 10% EtOH/90% corn oil, Sigma) at 100 mg/kg body weight once per diem for a period of five days. All stab injury and stroke surgeries were performed after a washout period of 14 days following the end of TAM administration. Control animals received no injury or stroke but underwent the same 4-OH TAM induction protocol as animals in each injury and stroke group.

### Cortical Stab Injuries

Ten *Foxj1*^*creERT2*^ mice at P1 and eight at P39 were induced by Tamoxifen (TAM, 75 mg/kg body weight) administered intraperitoneally for five consecutive days either directly or to females with newborn pups. Both groups were anesthetized by isofluorane at P42 and received bilateral stab wounds in their motor cortices (1 mm lateral to Bregma) using a 0.5 mm-tipped sterile stainless steel probe mounted on a stereotaxic apparatus. Mice were then sutured along the scalp, removed from the stereotactic frame and allowed to recover. Mice were sacrificed 14 or 30 days later by Avertin overdose followed by intracardial perfusion.

### Stroke Models

Focal photothrombotic (PT) cortical strokes were produced as previously described^19^. Mice were placed in a stereotactic frame under isoflurane anesthesia and the skull was exposed through a midline incision. A cold light source (KL1500 LCD; Carl Zeiss MicroImaging) attached to a 40× objective provided a 2 mm diameter illumination spot and was positioned +1.50 mm lateral to bregma to produce a 2 mm diameter focal stroke upon illumination. Rose Bengal (10 mg/ml, Sigma-Aldrich) was administered via i.p. injection at 100 mg/kg body weight. After 5 min, the brain was illuminated through the intact skull for 15 min. Mice were then sutured along the scalp, removed from the stereotactic frame and allowed to recover. Body temperature was maintained at 37.0 °C with a homeothermic warming system (Kent Scientific) throughout the procedure.

Middle cerebral artery occlusion (MCAO)-induced cortical barrel field strokes were produced by occlusion of a distal branch of the middle cerebral artery as previously described^10^ with modifications. In this model, ischemic cellular damage is localized to somatosensory and motor cortex. Under isoflurane anesthesia, mice were placed in a stereotaxic frame and the skull was exposed through an incision between the eye and external auditory meatus. A small burr hole was drilled in the skull to access and permanently occlude a distal branch of the MCA using a small heater probe. The surgical site was sutured and mice were then removed from the stereotaxic frame and placed in a supine position. A midline incision was used to expose the ipsilateral common carotid artery, which was permanently occluded using a small heater probe. The surgical site was sutured and mice were allowed to recover. Body temperature was maintained at 37.0 °C with a homoeothermic warming system throughout the procedure.

Ischemic strokes limited to the striatum were produced by injection of the endothelial nitric oxide synthase (eNOS) inhibitor N5-(1-iminoethyl)-L-ornithine (L-NIO) as described^20^ with modifications. Under isoflurane anesthesia, mice were placed in the supine position and a midline incision was used to expose the common carotid arteries adjacent to the trachea. The ipsilateral common carotid artery was permanently occluded using a small heater probe. The surgical site was sutured and mice were placed in a stereotaxic frame. The skull was exposed through a midline incision and a small burr hole was drilled in the skull at coordinates of +0.50 mm anterior and +2.75 mm lateral to bregma. A total of 4 µl L-NIO (27 mg/ml, Millipore) was injected with a microliter syringe (Hamilton) through cortex and white matter into the striatum, delivering one-third of the 4 ul total volume of L-NIO at coordinates of −3.00 mm, −2.60 mm, and −2.20 mm ventral to bregma. Each injection was performed at 200 nl/min using a small volume syringe pump (Chemyx) attached to the stereotaxic frame. The surgical site was sutured and mice were allowed to recover. Body temperature was maintained at 37.0 °C with a homeothermic warming system throughout the procedure.

### Immunohistochemistry, Imaging, and Cellular Quantifications

Mice of defined experimental end points were perfused with 4% Parafomaldehyde and the brains were removed from the skull and post-fixed overnight. Brains were sectioned into 50 µm sagittal sections with a vibratome. For immunohistochemistry, floating brain sections were blocked with 10% goat serum and 1% Triton-X 100 in 0.1 M PBS for 1 hour at room temperature. Sections were then incubated at 4 °C overnight with one or a combination of the following antibodies: Rabbit anti-RFP (Abcam, ab6231; 1:1000), Rabbit anti-GFAP (Millipore, MAB360; 1:1000), and Rat anti-GFAP (Life Technologies, 13-0300; 1:500), Guinea pig anti-DCX (Millipore, AB2283; 1:1000), Rabbit anti-Olig2 (Millipore, AB9610, 1:1000), Mouse anti-TuJ1(Biolegend, 801201, 1:500), Rabbit anit-S100 (Dako, 20311; 1:1000), Mouse anti-Neun (Millipore, MAB377; 1:1000). DAPI was used counterstaining the brain sections. All primary antibodies were dissolved in PBS with 1% goat serum, 0.3% Triton X-100. Sections were washed in PBS three times (five minutes each), followed by incubation with species-specific conjugated fluorescence secondary antibodies for one hour at room temperature. After secondary antibody incubation, the sections were washed with the same washing protocol and coverslipped. Sections were imaged on a Nikon Eclipse EZ-C1 or Olympus FV1000 confocal microscopes.

*Foxj1*^*creERT2*^ labeled tdTom+ cells in various forebrain structures in naïve and experimental mice were counted in tile-scanned confocal images of sagittal sections from three mice in each group (five sections containing intact olfactory bulbs, 20× objective, 1024 × 1024 resolution, 0.62 µm per pixel). DAPI counterstain was used to delineate architectonic boundaries using the Allen Brain Atlas P56 mouse sagittal reference panels (http://atlas.brain-map.org/atlas?atlas=2). Counting was conducted in ImageJ using the Cell Counter plugin (ImageJ, US National Institutes of Health). Neuronal, glial, and unidentifiable cells were classified based on morphology or marker co-labeling (Fig. 2). Cell counts were transferred to Microsoft Excel for statistical analyses. Total tdTom+ numbers presented in Figs 1e, 3e, 4e, 5e and 6e are mean ± s.e.m of cells counted in various regions from five sections per animal. Percentages of cell types presented in Figs 1f, 2c, 3f, 4f, 5f and 6f were calculated for each animal form the same sections and images, and presented as mean ± s.e.m calculated from three animals per experimental condition. Significance was determined by 2-tailed t test at p < 0.05.
